# Nurse and Other Healthcare Managers' Experiences and Recommendations for Patient Incident Reporting Processes and Real‐Time Software Development: A Qualitative Study

**DOI:** 10.1111/jan.70220

**Published:** 2025-09-18

**Authors:** Saija Koskiniemi, Laura Jukarainen, Tiina Syyrilä, Elizabeth Manias, Katri Hämeen‐Anttila, Marja Härkänen

**Affiliations:** ^1^ Department of Nursing Science University of Eastern Finland Kuopio Finland; ^2^ Monash Nursing and Midwifery Monash University Melbourne Australia; ^3^ School of Pharmacy University of Eastern Finland Kuopio Finland; ^4^ Research Centre for Nursing Science and Social and Health Management, Kuopio University Hospital, Wellbeing Services County of North Savo Kuopio Finland

**Keywords:** focus group, incident reporting, patient safety, risk management

## Abstract

**Aims:**

To (1) analyse managers' experiences with handling patient safety incident reports in an incident reporting software, identifying key challenges; (2) analyse the incident report processes from the managers' perspective; (3) examine managers' perceptions of ways to support and improve health professionals' experiences of report‐handling processes; and (4) investigate how, from their point of view, incident reporting software should be developed in the future.

**Design:**

A descriptive qualitative study.

**Methods:**

Interviews and focus group discussions on Microsoft Teams from 11/2024 to 3/2025, including 16 participants, analysis with deductive and inductive content analysis.

**Results:**

Of 16 participants, 15 were managers and one was a patient safety expert. Most were nurse managers (*n* = 9). Four discussion themes were divided into 30 categories. Participants highlighted the need to improve the reporting software's terminology, classification and analysis tools. The use of artificial intelligence was desired but not currently integrated into the software. Participants were unsure of their skills to use all the software features. Clear and transparent handling processes, feedback, managers' behaviour and communication methods were seen as key to improving staff's experience with report processes. A real‐time warning system was considered beneficial for various incident types. Specific questions must be answered before further developing such systems.

**Conclusion:**

This study deepened the understanding of reporting software's challenges regarding its handling features. The handling processes of incident reports had multiple shortcomings, which may negatively affect health professionals' experiences in report handling. Real‐time warning systems could assist healthcare managers in processing reports.

**Implications for the Profession and/or Patient Care:**

Organisational‐level guidance for incident report processing is needed. Improvements to report processing and reporting software can improve shared learning and understanding of the status of patient safety.

**Patient or Public Contribution:**

No patient or public contribution.

**Reporting Method:**

COnsolidated criteria for REporting Qualitative research Checklist.

## 1 Introduction

The ultimate purpose of reporting patient safety incidents is to understand what went wrong, learn from previous mistakes, and reduce the harm caused during treatment by preventing the recurrence of incidents (World Health Organization 2020). To achieve these purposes, it is essential that incident reporting software includes all necessary features for the effective handling of reports and that the handling processes facilitate shared learning. When investigating the development needs of electronic patient safety‐related incident reporting software, it has been found that users are particularly dissatisfied with the features related to report processing (Koskiniemi, Syyrilä, Hämeen‐Anttila, Mikkonen, et al. 2024). Efficient and comprehensive handling of incident reports is a key part of the reporting process; reporting incidents alone does not prevent similar incidents, nor does analysing reports lead to learning from them (Macrae 2016).

## 2 Background

Health professionals' perceptions of incident reporting are under‐studied and studies have focused mainly on nurses' perceptions (Koskiniemi, Syyrilä, Hämeen‐Anttila, Manias, and Härkänen 2024; Napoli 2022). The incident report handlers, often unit managers, are dissatisfied with the incident reporting software handling features (Koskiniemi, Syyrilä, Hämeen‐Anttila, Mikkonen, et al. 2024). In Finnish healthcare, unit managers are usually nurse managers or physician managers; nurse managers handle reports made by nursing staff and physician managers handle reports made by physicians. Responsibilities of incident report handling vary in different countries. For example, in Estonia, unit managers are responsible for analysing reports and planning actions (Uibu et al. 2023). In Japan, the responsibility falls on a patient safety manager (a nurse practitioner) (Kodate et al. 2022). Nurses have the most significant responsibility for incident reporting in care homes (Scott et al. 2025) and hospital settings (Goekcimen et al. 2023). Therefore, the role of nurse managers as report handlers has become more pronounced compared to other health managers.

The actions needed after an incident are not straightforward (Liukka et al. 2020). When the number of incident reports is high, it poses a challenge to managers (Liukka et al. 2019). A previous study demonstrated that valuable data collected through incident reporting has been utilised in various ways in the care home context, such as identifying staff's training needs and generating summaries and visual outputs for noticing trends and needs for practice changes (Scott et al. 2025). Current incident reporting software does not support unit managers to achieve the purposes set for incident reporting; the quality of reports is often poor (Macrae 2016), handling features in software are inadequate (Koskiniemi, Syyrilä, Hämeen‐Anttila, Mikkonen, et al. 2024), and the overall design, accessibility, classification, anonymity and tracking possibilities need improvements (Koskiniemi, Syyrilä, Hämeen‐Anttila, Manias, and Härkänen 2024). Finally, recommendations are rarely stated in the incident reports (Liukka et al. 2019). Handling features, such as combining summaries and visual outputs, analysing root causes, investigating trends and asking for additional information from the reporter (Koskiniemi et al. unpublished), could help to process the incident reports effectively. However, in this era of technology, an ideal electronic incident reporting software would support efficient report handling and enable managers to focus on actual actions following the reporting process.

Systems that automatically and in real‐time identify critical factors from incident reports and warn about them so that, if necessary, support for the personnel involved in the incident can be initiated immediately after the report is submitted do not exist internationally. A unique system that identifies critical factors from patient records, such as patients returning to the emergency department within 24 h of discharge from surgery, and alerts the quality control department has been developed in India (Hospital Management Asia 2023). In Finland, incident reporting software for patient safety‐related incidents does not currently screen the reports nor alert on urgent or severe cases in real‐time. It can take weeks, even months, from submitting a report to processing it (Central Uusimaa Wellbeing Services County 2022; SiunSote 2023). Understanding the managers' perceptions is necessary to support the future development of a real‐time warning system in incident reporting software.

Patient safety incident reporting software can be national or local, hospital‐wide or speciality‐specific, and its features may vary. However, a previous study has shown that incident reporting software faces similar challenges globally (Koskiniemi, Syyrilä, Hämeen‐Anttila, Manias, and Härkänen 2024) The World Health Organization published the Minimal Information Model for Patient Safety Incident Reporting and Learning Systems in 2020 to address global issues such as unstandardised data collection and to support learning from incidents (World Health Organization 2020). Based on our literature search, handling features of incident reporting software have not been previously studied from the perspective of those who process the reports. Furthermore, Finland is currently undergoing a national structural reform of incident reporting software, led by the Ministry of Social Affairs and Health (2022). This study offers transferable insights and a valuable starting point for international discussion on improving the functionality and usability of incident reporting systems from handlers' perspectives.

## 3 Study

This study aims to (1) analyse managers' experiences with handling patient safety incident reports in an incident reporting software, identifying key challenges; (2) analyse the incident report processes from the managers' perspective; (3) examine managers' perceptions of ways to support and improve health professionals' experiences of report‐handling processes; and (4) investigate how, from their point of view, incident reporting software should be developed in the future.

## 4 Methods

### 4.1 Design

The study employed a qualitative descriptive approach. Qualitative research methodology is suitable when studying topics that have not been widely studied (Hennink 2014). Originally, data collection was planned to be conducted utilising FGDs only. FGD is a suitable method for collecting experiences and perceptions on a specific topic from individuals with a similar background or experience (Hennink 2014). The study followed the FGD process described by Morgan et al. (1998), supplemented with guidance from (Hennink 2014), particularly in adapting the method to online discussions.

However, due to the limited number of participants, the approach was adapted to include individual interviews and dyadic interviews in addition to FGDs. This flexible design enabled us to incorporate diverse perspectives while maintaining the depth and richness of the data. The data were analysed using deductive and inductive content analysis. Inductive content analysis is a suitable method for analysing phenomena that have not been studied in detail (Kyngäs et al. 2019) and was used to identify categories. However, analysis was supplemented with deductive analysis (Kyngäs et al. 2019) using themes identified in the previous study (Koskiniemi et al. unpublished). In reporting this study, the Consolidated criteria for Reporting Qualitative research (COREQ) checklist was used (Supporting Information S1; Tong et al. 2007).

### 4.2 Study Setting and Recruitment

Participants were recruited via email from two wellbeing services counties in Finland by the target organisations' contact persons, not by the research team. One of the five Finnish university hospitals is located in these wellbeing services counties. Wellbeing services counties have been responsible for organising public health care, social care services, as well as rescue services within their areas since 2023. There are 21 wellbeing services counties in Finland, and their boundaries mostly follow the regional division. However, the region of Uusimaa is divided into four counties, and the City of Helsinki organises services independently. (Ministry of Social Affairs and Health 2025) In Finland, two electronic patient safety incident reporting software, which are rather similar in terms of content, are used broadly in wellbeing services counties. The target wellbeing service counties used those reporting software. In total, there were approximately 30,000 employees in the target wellbeing services counties. All incident report handlers, mostly unit managers, from all units of the wellbeing services counties' social services and primary and specialised healthcare units were approached, as total population sampling was used.

### 4.3 Inclusion Criteria

The inclusion criteria required that the person handle patient safety incident reports using electronic incident reporting software. Thus, participants had backgrounds in nursing, medicine and social work. Discussions were held in Finnish; therefore, participants had to have sufficient Finnish language skills.

### 4.4 Data Collection

Initially, those who wished to participate in this study provided their background information via an electronic Webropol form. They also chose possible discussion sessions from the pre‐scheduled times. The research team contacted all individuals who had completed the form via email and agreed on a date for the FGD. Also, the interview guide, including discussion themes, was sent to the participants in advance (Supporting Information S1). Seven of those who completed the background questionnaire did not participate in any FGDs. Some of them were not reached via email, and some could not participate due to their busy work schedules.

The original plan to collect the data through FGDs was modified due to a limited number of participants. Due to last‐minute cancellations, one group was converted to an individual interview, and two groups were converted to dyadic interviews. Each participant (*n* = 16) engaged in one interview or FGD during their work hours. Participants were divided into homogeneous groups based on their occupational category: physician and dentist managers were grouped, as were nurse managers and social service managers. Homogeneity between participants was justified to ensure open discussion as participants are more likely to share their experiences and perceptions with others having a similar background (Hennink 2014). An individual interview, two dyadic interviews, and four FGD groups were held from November 2024 to March 2025. Interviews and discussions lasted between 34 and 86 min, with an average duration of 59 min.

Interviews and discussions were conducted via Microsoft Teams and recorded with participants' permission using Teams' recording feature (audio and visual recording), with a backup recording made using an audio digital recorder. In all groups, the interviewer (SK) was an MSc, and she was working as a doctoral researcher. The observer (LJ) in most groups was a master's student, and she was working as a research assistant. In one group, the observer (TS) was a PhD, and she was working as a postdoctoral researcher. All of them were working at the same university, were from the field of nursing science, and were registered nurses. The observer did not participate in the individual interview but did participate in each dyadic interview and FGD. None of them knew the participants or had worked in the target organisations or had experience with the FGD method. The research group included a researcher (KHA, professor and PhD in Pharmacy) with comprehensive experience in using the FGD method, with whom discussions were held about the practice of the FGD method. All interview and discussion sessions followed a discussion guide developed based on the results of the previous survey (Koskiniemi, Syyrilä, Hämeen‐Anttila, Mikkonen, et al. 2024). The guide was piloted with the first FGD group, and no changes were made. The pilot FGD was included in the data. At least one supporting question related to each discussion theme was posed during every interview and discussion group (Supporting Information S1). Although the interactional nature of FGDs differs from interviews, the same semi‐structured guide was used across the sessions to ensure consistency. The observer took notes during each FGD and dyadic interviews, and the interviewer took notes during an individual interview. New FGDs were organised until new groups provided little new information on this study's interests.

### 4.5 Data Analysis

**FIGURE 1:**
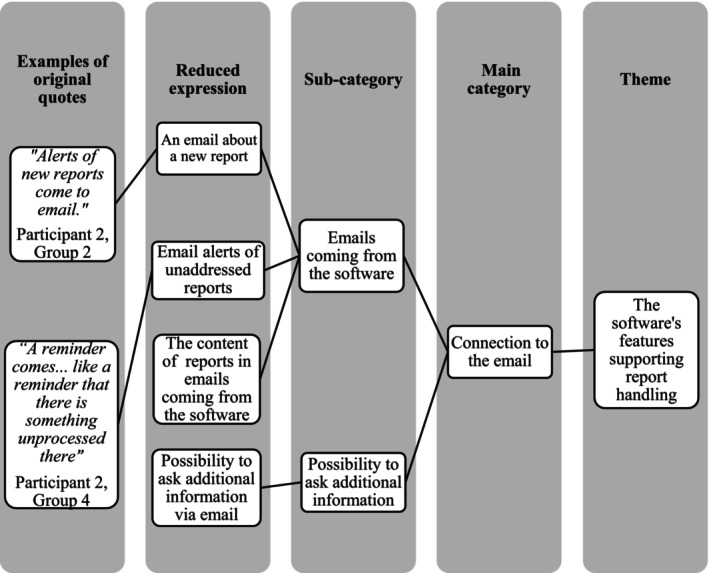
An example of content analysis.

Audio records were transcribed verbatim by the interviewer within 24 h after each group. The observer checked the transcriptions, but they were not returned to the participants for comments. The final transcribed data comprised 78 pages (Arial, 12‐point font, 1.5 spacing). The background questions were analysed using R software version 4.2.2 (R Core Team 2021). The interview and FGD data were manually analysed using deductive content analysis on the theme level (Koskiniemi et al. unpublished) and inductive content analysis to identify categories (Kyngäs et al. 2019). The transcribed data was carefully read through several times. The first author (SK) identified, collected and coded meaning units (parts of a sentence, a sentence, or multiple sentences) that addressed the research questions (Figure 1). The data analysis was conducted in Finnish, and quotes were translated into English by the first author. The first five groups were analysed first, and the last group was analysed using the existing coding frame. Other research group members (LJ, TS and MH) reviewed and commented on the analysis. Additionally, the result text was emailed to participants for review and comment. Minor adjustments were made based on the comments of one participant. Finally, the number of groups mentioning a finding under each main category was calculated. Participants' non‐verbal communication was not considered in the data analysis.

Participants' experiences and perceptions were deductively collected under four themes: (1) the software's features for handling reports, (2) handling processes, (3) ways to improve health professionals' experiences of handling processes and (4) possible future developments in reporting software.

### 4.6 Ethical Considerations

This study followed ‘The ethical principles of research with human participants and ethical review in the human sciences in Finland’ guideline published by the Finnish National Board on Research Integrity TENK. The participants in this study were adults who gave their consent to participate, and their physical integrity was not compromised. Data collection posed no risks to the participants' mental wellbeing, did not include exceptionally strong stimuli and presented no safety threats to the participants. Therefore, an ethical review statement was not needed according to the Finnish National Board on Research Integrity TENK's guideline (TENK 2019, 62) The target organisations' contact people emailed the study's cover letter to all potential participants. Participants were informed of their rights in the cover letter and its attachments. The attachments were a privacy statement, an announcement of the study, and a link to a video that described the study's content. Potential participants could request more information about the study by contacting the first author via phone or email. Participation was voluntary and could be discontinued at any time. In the Webropol form, all participants provided consent to participate in the study and to have their discussions recorded. Audio and video records were deleted after careful transcription and verification of the transcriptions. The participant names were pseudonymised, and a code was given to each participant in the transcriptions (e.g., participant 1; group 2). Thus, there were no names or other potential identifying data in the transcriptions. The results are presented at the group level, and individual participants are not identifiable.

### 4.7 Rigour and Reflexivity

The interviewer and the observer discussed their thoughts after each FGD to share insights and gain a shared understanding of the general content of the FGD. The observer reviewed the transcriptions, and participants reviewed the result text, which increased the study's credibility. The atmosphere during discussions was confidential. Participants openly shared their own mistakes and insecurities about their work and asked other participants for advice on problems they faced in using incident reporting software or handling the reports. This study's context and participants' characteristics were described in detail to facilitate the assessment of transferability. Altogether, the method used, data collection and analysis were described thoroughly. Reflexivity was done throughout the research process (Busetto et al. 2020). The research group discussed the analysis process, and the researchers' background, including their involvement in FGDs, was disclosed to the participants. Based on the interviewer's previous studies, the interviewer formulated an opinion on the research topic. However, the interviewer did not express their thoughts during FGDs.

## 5 Results

FGDs consisted of three to five participants. The first FGD was converted to an individual interview, and two groups were converted to dyadic interviews due to last‐minute cancellations (Table 1).

**TABLE 1:** Participants (*n* = 16) in FGDs and interviews.

The number of the interview or FGD	Nurse managers	Social service managers	Physician or dentist managers	Patient safety manager or a patient safety specialist (nursing background)	A total of participants in each session
1		1			1
2	4	1			5
3	2	1			3
4			2		2
5	3				3
6				2	2
A total of participants' occupational groups	9	3	2	2	16

Over half the participants were nurse managers (*n* = 9; Table 2). The participants' average work experience in health or social care was 19 years, and most of them (*n* = 9) had been handling incident reports using electronic software for over five years. Only one of the participants was not a manager; however, the participant was a patient safety expert responsible for handling incident reports.

**TABLE 2:** Demographic characteristics of participants (*n* = 16).

Characteristics	Range/mean	*n* (%)
Age	33–62/44	
Gender		
Female or not prefer to say		15 (94)
Not prefer to say		1 (6)
An occupational group whose manager		
Nursing staff		8 (50)
Social service staff		3 (18)
Physicians or dentists		2 (13)
Nursing, social service and rehabilitation staff or another manager		2 (13)
Not a manager, but handles incident reports		1 (6)
Working area		
Primary healthcare		6 (37)
Social services		5 (31)
Special healthcare		2 (13)
Primary and special healthcare		1 (6)
In all the above		2 (13)
Work experience in social or healthcare (years)	5–35/19	
Experience in handling incident reports using electronic patient safety incident reporting software (years)		
Under 1		2 (13)
1–2		4 (25)
3–4		1 (6)
5–10		6 (37)
Over 10		3 (19)

**FIGURE 2:**
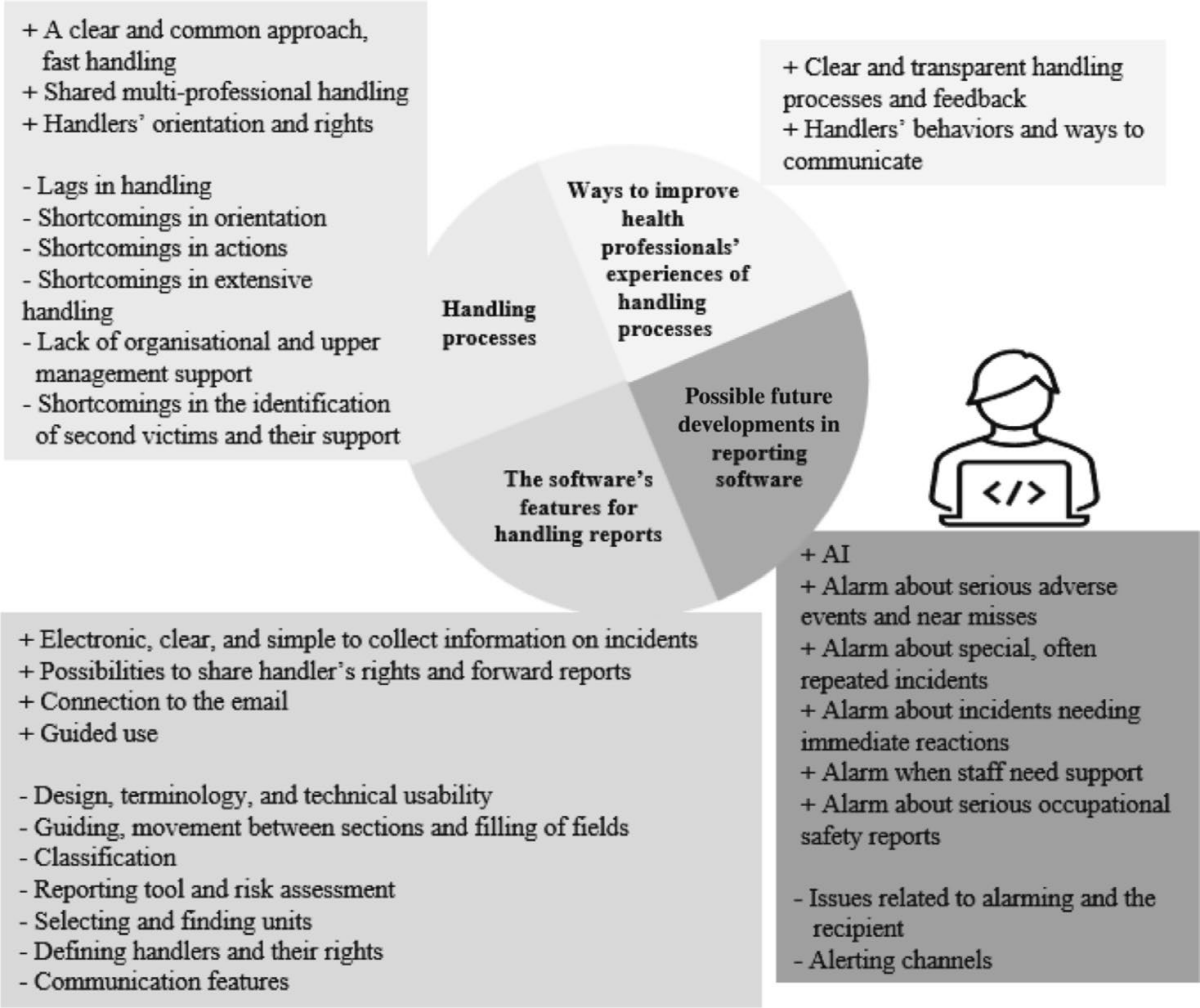
The main categories of incident report managers' experiences and perceptions regarding the software's features for handling reports, reporting processes, improving health professionals' experiences of handling processes and possible future developments in reporting software.

Handlers' perceptions and experiences were discussed under four discussion themes. Figure 2 shows the main categories of incident report managers' experiences and perceptions regarding four themes: the software's features for handling reports, reporting processes, improving health professionals' experiences of handling processes and possible future developments in reporting software.

### 5.1 The Software's Features for Handling Reports

Some participants found that the software helped them in processing reported incidents. Other participants found that it challenged them. They were satisfied that incident reporting and handling is possible using electronic, clear and simple software (mentioned in 3/6 groups), which enables the collection of information on patient safety incidents in one place.

A wide range of information is gathered in one place, allowing for the documentation of various types of incidents. (Participant 1; Interview 1)

Participants noted that the software's connection to the email system (mentioned in 3/6 groups) facilitated handling. They could receive notifications about new reports, including part of the report's content. The software also sent an email when the report's handling progressed. The software enables handlers to request additional information about the incident, which is then forwarded to the reporter's email. Participants perceived these features as supporting their work.

But the fact that it comes in an email means I can see it quickly. It gives you a sense of whether you must react to it right away or continue it the next day or the following week. (Participant 3; Group 3)

Participants acknowledged how the software guided its use (mentioned in 3/6 groups) through filling out the form and using the software. Guides were short texts in the software explaining what needed to be filled in the fields. Participants valued the feature that guided reporters to choose whether to report under their own name. The software guided handlers to focus on processes rather than individuals.

Guiding. Therefore, the necessary information is provided without considering what else should be included in the reports. The software guides progress correctly and opens new information and boxes by clicking. (Participant 3; Group 2)

The possibility of sharing a handler's rights and forwarding reports (mentioned in 2/6 groups) to different units and upper management supported the handlers' work. Reports could be forwarded to Excel, and handlers frequently utilised this option. If the handler continued through the entire handling process in the software and clicked the correct box, the information from the handling process was forwarded to the reporter.

For example, I can forward it to the health and safety representative or our upper management. I mean, this kind of multi‐professional opportunity that this incident reporting software provides. (Participant 2; Group 5)

Participants mentioned software features that challenged report handling more than those that supported it. The software's design, terminology and technical usability (mentioned in 5/6 groups) presented challenges. The software kept the reports' processing status uncompiled, even if the report had been waiting for additional information from reporters for a very long time. The software was challenging to navigate, and the overall design should be updated from the participants' perspectives. The terminology did not account for all environments where it is used, and was primarily focused on the healthcare and hospital context.

The software uses the term ‘patient room’, which is very healthcare‐oriented. We have clients and a homely lifestyle here, so the terminology in our everyday lives is very different, and incidents are different. (Participant 1, Interview 1)

Based on participants' perceptions, the way reporting forms guide reporters, movement between sections and filling in fields (mentioned in 5/6 groups) need improvements. Movement between sections in the software was frustrating when it took considerable time. The form also made annoying moves on the screen after clicking boxes. Participants said they often needed to write the same text in multiple places within the software. Also, handlers' texts in the software between similar incidents were often almost identical. Therefore, participants hoped for drafts of typical incidents to be ready. For example, if a typical incident occurred, such as a medication error, the handler would choose a ready draft as a basis for report processing when handling the incident. The draft would include prepared text in the field, like actions made, and the handler could modify the text if necessary. Although some participants mentioned that the software's method of guiding users supports handlers' work, a lack of guidance was also perceived as a challenge. The software accepted incomplete reports when it should have guided reporting with supporting questions and required fully filled reports. Incomplete reports took the handlers' time when information that the reporter would have known was missing.

It allows reporters to answer so broadly that I find it a bit difficult. And then, on the other hand, it asks such specific questions that I wonder if this is essential now. (Participants 2; Interview 4)

The classification (mentioned in 4/6 groups) was challenging in multiple ways. Technically, it was troublesome to find the correct sorting from very long dropdown lists. Participants assumed that reporters often made their classifications incorrectly, partly because of this. Incorrect classification made statistics untrustworthy when similar incidents were not classified in the same way. Participants stated that the classification was not broad enough to encompass all types of incidents. For example, classification did not fit for processing near‐misses.

It somehow guides that now has happened some catastrophe, but when we have this kind of near misses, they do not fit the classification. (Participant 2; Interview 4)

A poor reporting tool and risk assessment matrix (mentioned in 4/6 groups) caused double and manual work for participants. Handlers must produce statistics for various stakeholders; however, the software did not enable them to export all the types of statistical reports they needed. Statistical reports do not include all the necessary information, and it is not possible to select which fields the handler wants to export to the report. Participants desired more comprehensive statistics, such as the possibility of cross‐tabulation, rather than just the frequencies of reported incidents. Participants said that the risk assessment matrix for assessing the incident's severity and likelihood of recurrence should be clearer and include a usage guide.

I decided to export all statistical reports from all 10 units. It meant that I must separately click in that damn ‘software's name’, that now I want adverse events from (the unit's name) and then near misses from the same unit. I needed to do this from all 10 units because it was not possible to export all reports at once. (Participants 1; Interview 4)

Wellbeing services counties are big organisations, including hundreds of healthcare and social services units. Based on participants' perceptions, selecting and finding the correct unit (mentioned in 4/6 groups) related to the incident or forwarding the report could be challenging for reporters and handlers. The software did not include the possibility to search by typing the unit's name. The lists of different units were extremely long, and units were not always written the same way in the reporting software and the organisation's intranet.

If you want to transfer or copy the report to another unit, finding it (the unit) is quite challenging. (Participant 3; Group 5)

Defining handlers and their rights (mentioned in 3/6 groups) was unclear to the participants in the software. Handlers' rights did not expire automatically and could not be put on hold. The software did not include a feature to mark a specific handler in the report.

There should be a tagging system in the software, so that now this and that physician or chief physician must handle this issue. (Participant 3; Group 5)

Communication features (mentioned in 2/6 groups) could be improved in many ways in the software, based on participants' experiences. Handlers could request additional information from reporters; however, flexible messaging between handlers and reporters was not possible.

I have sometimes wondered about something like that too, that the feedback…the feedback section is poor. Or maybe I just do not know how to use it. It could be the user's fault. (Participant 2; Group 5)

### 5.2 Handling Processes

Participants shared their experiences of the incident report‐handling processes. Participants were satisfied with their experiences of shared multi‐professional handling (mentioned in 4/6 groups). They said reports were discussed regularly during team meetings to identify preventive actions. From a quality perspective, some units had regular work groups where reports were also discussed. Participants shared their positive experiences about discussing reports with upper management.

There are a physician and a nurse representation, and if necessary, others are invited to that meeting. The reports are handled monthly. (Participant 3; Group 5)

Handlers' orientation and rights (mentioned in 4/6 groups) supported incident report handling when multiple handlers could share responsibilities. Handlers' rights meant the right to see the unit's incident reports, as well as process them. This helped in cases of absences. Not only managers but also non‐manager nurses could have a handler's rights. For some individuals, such as a nurse responsible for medicines, it was possible to give access to specific types of reports. A nurse responsible for medicines takes care, for example, of the unit's pharmacotherapy plan, which contains the guidelines for the pharmacotherapy process. Participants said a thorough orientation to the handler's work helped them learn to handle incidents. Organisations also provided various materials for learning the use of the software.

There are named handlers, not only managers, but also nurses in charge. So, the handling responsibilities have been shared with others as well. (Participant 2; Group 6)

Positive experiences were also related to a perceived clear and common approach and fast handling (mentioned in 3/6 groups). Unit‐level approaches were especially seen as workable.

In my opinion, our process works quite well at the unit level. (Participant 3; Group 2)

On the other hand, participants also expressed multiple concerns regarding the handling processes. For example, lags in handling (mentioned in 4/6 groups) were due to the handlers' workloads and challenges in finding time to handle reports within a workgroup. Between occupational groups, there were differences in handling lags. Participants had experiences where, in some units, reports were always handled on time, while in others, reports could remain unhandled for two years. The handling tended to accumulate at the end of the year, and when the unit got a new manager. The new manager's position was challenging, as the previous manager may not have addressed all the reports submitted before the change.

I had to handle dozens of incident reports there…Unhandled reports from the time before I even was there, because my position was moved there. Issues I knew absolutely nothing about. (Participant 2; Group 3)

Deadlines are strict, and you should handle reports within a certain time frame. So then I cannot mark it ‘ready’ without kind of lying that yes, this has now been done according to this protocol, even though I haven't had time actually to open and read it at this very moment. (Participant 3; Group 2)

A lack of organisation and upper management support (mentioned in 4/6 groups) was described. There was a lack of a standard handling model. Therefore, participants perceived a lot of variation in handling processes within an organisation. Their perceptions were that good practices should be shared within an organisation. Participants also experienced upper management not reacting to reports that handlers had forwarded to them. Participants said they usually did not get an answer from upper management.

And then I also use that, that I forward it to a higher level for handling. But I have never received a single answer from there. (Participant 1; Group 3)

Shortcomings in orientation (mentioned in 3/6 groups) led to participants needing to learn how to handle reports independently. It also led to a lack of awareness of the software's features and insecurity about their knowledge and skills in using it.

I have not received any kind of training on this. (Participant 1) Yes, that is it. Learning the hard way. (Participant 2; Interview 4)

Shortcomings in actions (mentioned in 3/6 groups) revealed concerns that they did not have time to plan and implement actions based on the reports. The planning of actions was inadequate because participants were unsure who could assist them. Participants also found that planning actions based on individual reports was difficult. Participants were unsure whether the reporting and report processing had an effect.

Sometimes it feels like they are just left there…That the report is made, and I handle it and…and…The chief physician handles it, or we look at them together, but then nothing happens after that. (Participant 2; Group 5)

Shortcomings in extensive handling (mentioned in 3/6 groups) meant that participants felt handling was only an assessment of the individual handler. Participants said that no one reviewed the work of the handlers. Handling stayed at the unit level.

We do not have any organisation‐based model for how we should handle reports. (Participant 1; Interview 4)

Participants expressed that the identification of second victims and their support (mentioned in 1/6 groups) should be better organised. Participants hoped that occupational healthcare would join in report‐handling processes that ensure healthcare professionals involved in incidents receive the support they need. Currently, supporting healthcare professionals lies on the handlers' shoulders. Participants noticed that even a couple of decades after being involved in an incident, some health professionals become emotional when they speak about what happened.

Even occupational health could be involved in this process, because if something bigger happens… I do not know how employees think about incidents at home and grieve about what happened. Even though they are completely human mistakes. So, in that process, occupational health could be involved. (Participant 2; Group 5)

### 5.3 Ways to Improve Health Professionals' Experiences of Handling Processes

Based on participants' perceptions, clear and transparent handling processes and feedback (mentioned in 3/6 groups) could support health professionals' experiences handling incident reports. Participants noted that handling processes should be transparent, with all steps clearly outlined for health professionals. Participants also revealed that they did not always have time to handle reports, so they would only mark them as ready without taking any action. There were also concerns that incident reporting had not improved the situation, with, for example, patient falls even after years. Participants hoped to be able to thank reporters and provide feedback on the report, including how the handling progressed and what actions were taken.

Feedback would encourage them to do more reports if they got good feedback about it. Thanks, and the report has progressed, and if it led to something, or where the report will go next. Maybe this would encourage them more. And would remain that we are not really looking for the guilty, but we want to correct practices. (Participant 2; Group 5)

Participants said the handlers' behaviour and communication methods (mentioned in 2/6 groups) could support the health professionals' experiences. The blameless handling of incident reports required a lot of effort from handlers. They thought they needed to clearly express that they were not looking for guilt and, in doing so, create a safe and open culture of dialogue. Participants noticed that they need to actively work to ensure that health professionals trust that nothing negative will happen to them after reporting. This was due to the possible negative ways previous managers handled incidents. Participants noted that handlers must clarify the meaning of reporting and correct any possible misconceptions about handling. Communication regarding incident reports needs to be well‐planned and neutral. Participants said that if the report concerns an individual employee's action, a shared discussion about the incident is probably unnecessary. Sometimes, reports were made to cast a negative light on a particular employee, often based on personal opinions or issues with that individual. Inappropriate reports should be corrected by the handlers right away.

When I read the reports, many opinions come to mind. So, that I can then, however, be a neutral handler, is probably the most important thing. (Participant 4; Group 2)

### 5.4 Possible Future Developments in Reporting Software

Participants suggested how artificial intelligence (AI) could be utilised in incident reporting software to assist handlers. They saw the lack of AI (mentioned in 6/6 groups) as one shortcoming in the software's handling features. Participants suggested using AI to group similar incidents when human‐made classification is often inconsistent. For example, AI could assist in filling out the form by formulating text based on the handler's notes, performing classification and providing guiding advice. AI could connect the reporting software and organisations' systems for electronic guides.

Somehow, the software could give our organisation's instructions to me. So, if it (the report) were related to medication, it would somehow be able to pick up instructions related to it, because we have those kinds of instructions a lot in our organisation. It could send it to me, that we have this kind of instruction in our organisation, and then I could forward it to the employees. (Participant 2)

I also face often the situation that we already have an instruction for this, and I could link it to the reporter. (Participant 3; Group 5)

To develop a new type of real‐time warning system, participants considered that situations required an immediate alarm after the report was submitted. An alarm about serious adverse events and near misses (mentioned in 3/6 groups) is needed. These situations would be, for example, an adverse event or near misses which caused or could have caused patient death or a threat to patient health. Additionally, incident reports requiring immediate action (mentioned in 2/6 groups) should alert handlers. Those kinds of reports could be related to, for example, medicines, devices and human resources.

Issues related to the load, the high load. So that you could then try to get more staff or something to balance the load a bit. (Participant 4; Group 2)

An alarm was desired for specific or often‐repeated incidents, even if the incident did not require immediate action (mentioned in 1/6 groups). Participants also wanted the real‐time system to alert them when staff need support (mentioned in 1/6 groups), such as a debriefing regarding the incident.

Which requires debriefing. So, situations needing immediate contact with people involved in the situation. (Participant 5; Group 3)

The real‐time system could also alert to serious occupational safety reports (mentioned in 2/6 groups). These situations may arise when an employee has been exposed to a highly contagious disease, a serious situation that poses a threat to staff safety, or when there are threatening events or violence.

Occupational safety reports regarding something serious had happened or where a risk that something could have happened to personnel. (Participant 3; Group 2)

Participants discussed other aspects that need to be considered when developing a real‐time warning system, including issues related to alarming and the recipient of the alarm (mentioned in 3/6 groups). They thought it would be important to define alerting reports carefully and to determine who the alert is directed at and how they should respond to it.

The system already gives notifications of never events. So, it needs to be well considered what types of reports would be alerted in this new way, in addition to them. (Participant 2; Group 6)

Participants mentioned various possible alerting channels (mentioned in 6/6 groups). The most effective way in real‐time would be to get the alarm onto the handler's phone. There could be an app for that, or alerting could be integrated into some existing app. The alerting sound should be loud and different from other sounds. The alarm could be sent to the handler's email if a different alerting sound were available and the handler has an email app on their phone. Participants also stated that they would prefer a phone call from health professionals rather than being alerted via the reporting software after submitting the incident report.

Or is there an app where the alarm comes in, so that it appears on the phone screen, because if you need to get a real‐time alarm, you do not get it via email. (Participant 1; Interview 1)

## 6 Discussion

Managers' thoughts on the development of an AI‐based real‐time incident warning system increased new understanding. Findings indicate that significant improvements need to be made regarding the reporting software's handling features, as many issues in the reporting software hinder managers' work. Managers described the lack of a common approach for report handling throughout organisations, causing concerns about unsystematic handling processes. Creating a positive experience for reporters and other healthcare professionals when handling incidents requires active work from handlers.

Handlers described multiple software features that challenged their work as incident report handlers. While dissatisfaction with incident reporting software is not a new finding, and previously, handlers' perceptions of incident reporting software were recognised on a general level (Koskiniemi, Syyrilä, Hämeen‐Anttila, Mikkonen, et al. 2024), this study further deepened that knowledge. The purpose of reporting software from the point of view of the handlers is to enable the effective utilisation of reported data and learning from incidents, not only passively receiving reports (World Health Organization 2020). Therefore, improvements to the software that consider handlers' perceptions may enhance not only reporting behaviour but also effective use of collected data. Current issues with the reporting software have, for example, led to additional work for handlers. Improvements to the software are a financial question in the first place. However, one adverse event in the intensive care unit may result in healthcare costs up to $10,660 (Rincón López et al. 2024). Therefore, if the improved software saves handlers' time, leads to more effective report handling, and thereby reduces the number of incidents, the investment would be cost‐effective and prevent human suffering. In addition, more attention needs to be focused on the handlers' training. This study showed that, in some cases, managers' knowledge regarding incident reporting software features was limited. Therefore, managers might benefit from training in incident reporting software features.

This study revealed that some participants questioned the effectiveness of incident reporting in improving practice. If managers do not observe practice changes or trust the reporting process, health professionals may experience similar doubts. Unfortunately, the lack of visible action in response to incident reports is a reality (Liukka et al. 2019). Previous studies have also shown that health professionals who report incidents often have negative experiences regarding the management of incident reports (Woo and Avery 2021). Negative experiences, such as being blamed, naturally decrease health professionals' willingness to report. Moreover, the lack of visible actions and practice changes decreases health professionals' trust in the significance of reporting (Koskiniemi et al. unpublished). In a previous study, unit managers' perceptions of safety culture were more positive than those of other occupational groups (Liukka et al. 2021); however, in this study, incident report handlers, who were almost all unit managers, identified weaknesses in learning from incidents from their perspectives. They also expressed that there was not always enough time to handle reports correctly.

Although incident reporting software continues to evolve and requires further technical developments, its actual impact depends on whether the reported incidents can lead to concrete and evidence‐based solutions. However, currently, there is no evidence‐based solution to all patient safety incidents. Some critical patient safety issues, such as the assessment of suicide risk (Fröding 2022; Smith 2022) and fall risk (Strini et al. 2021), still lack effective evidence‐based solutions despite years of incident reporting of these safety incidents. When it is known that a reported incident may not always have an evidence‐based solution, the purpose and value of reporting, as well as the role of the report handlers, become ambiguous. This perspective helps explain the handlers' doubts about the effectiveness of incident reporting found in this study. In addition, this study revealed that some report handlers felt they lacked sufficient time to handle reports correctly, leading them even to mark reports as processed when they had not been completed. When the number of reported incidents is remarkable in some countries (Macrae 2016), and the reality is that handlers cannot solve all reported incidents (Fröding 2022; Smith 2022; Strini et al. 2021), these aspects justify the discussion of whether incident reporting should be limited and rather focus on specific patient safety incidents.

Healthcare first‐line managers have recognised the importance of their role in working with patient safety (Hedsköld et al. 2021), and this study supports these findings. When discussing ways to support reporters and other health professionals' experiences of report handling, participants pointed to their role as managers. Based on the participants' perceptions, communication and interaction with staff when discussing reported incidents should be carefully considered. Managers' open and sensitive communication skills are also essential when health professionals are involved in an incident. This study found that health managers recognise the importance of supporting health professionals after they are involved in incidents, consistent with a previous study (Järvisalo et al. 2024). However, managers believed that supporting these health professionals lies too heavily on the shoulders of first‐line managers and should be done in cooperation with occupational healthcare.

As part of this study's theme of developing an AI‐based real‐time warning system for reporting software, participants were asked to discuss their ideal reporting software. All groups immediately mentioned the use of AI. As new implications of AI are continually recognised in patient safety and risk management, its full utilisation will occur sometime in the future (De Micco et al. 2025). The results of this study emphasise the need for end‐user participation in future development efforts. The user's deep, practical understanding of the requirements for new technologies should be considered in future development. Close collaboration with end‐users can also reveal an unexpected range of uses for AI. In this study, managers suggested that AI could be utilised, for example, to connect incident reporting software with organisations' electronic guides. Nurses expressed a preference for oral reporting over formal writing (Woo and Avery 2021); AI could also provide a solution for this.

Much effort has been made to improve incident reporting; however, the primary focus has been on improving the quality of the reports and safety culture. The need for skilled professionals to analyse incident reports has been recognised (World Health Organization 2024), but there is little discussion of the requirements for the incident reporting software's handling features. The amount of preventable harm occurring in healthcare (World Health Organization 2024) shows that all tools need to be fully utilised so that healthcare organisations can provide safe patient care.

### 6.1 Strengths and Limitations

Participants had diverse occupational backgrounds in health and social care, but shared experiences related to the study's topic. Although most participants were nurse managers, this can be seen as a strength of the study, as most healthcare employees are nurses. Researchers who led the discussions had no previous experience with the method; however, they studied the methodology. Methodological discussions were conducted with the professor (KH‐A) with comprehensive experience using the method, and pilot interviews were utilised to reflect methodological issues. Data triangulation was used: the analysis of the first five groups was conducted first, followed by the analysis of the last group (Carter et al. 2014). The analysis of the last group confirmed that the initial analysis included all necessary sub‐ and main categories. The option to participate online may have encouraged participation when in‐person participation was not required (Hennink 2014). Online participation likely did not hinder participation among health professionals who are used to working with various technologies.

This study has some limitations. Although the aim was to use FGD for data collection, due to the limited availability of participants, some data were collected through individual and dyadic interviews. Despite this adjustment, the conducted FGDs had a limited number of participants within each focus group. None of the FGDs included the typical six to eight participants; there were three to five participants in FGDs. A limited number of participants in FGDs may reduce some of the interactive dynamics. (Hennink 2014) Regardless of the number of participants in each group, they were noted to interact with each other dynamically. The collected data provided rich and varied insights into an understudied topic. The data was sufficient to address this study's aims. Although physician managers represented a small sample, they complemented the rest of the data well. Most late‐minute cancellations were from groups of physicians. The interviews and discussions were conducted online, which limited the interviewer's possibilities to manage dominant and encourage quiet participants (Hennink 2014).

### 6.2 Implications for Policy and Practice

This study identified several areas for improvement in incident reporting and incident reporting software. Clear, transparent organisation‐level guidance for incident report processing and the implementation of such guidance would be essential. Currently, report handlers have recognised that there is much variation in how incident reports are processed. In the worst case, even report handlers do not believe that incident reporting improves patient safety, as some participants expressed in this study. Staff's negative experiences regarding how reports are discussed within units can affect their willingness to submit new reports, even decades after the negative experience. Clear guidance could improve the quality of report processing, promote shared learning from reported incidents, and increase reporting rates.

This study shows that several aspects of the handling feature of incident reporting software can be improved to better support report handlers in their work. Collaboration with software designers should not be limited to between designers and a specific patient safety expert. The development of such a system must be done in close collaboration with actual software users and, in this case, incident report handlers. Only this way is possible to ensure optimal features for processing reports. Incident reporting software is capable of doing much more than just storing reports: in this era of technology, those systems could include effective tools to analyse reports and support learning from them.

## 7 Conclusions

Incident reporting is essential for learning from errors in healthcare, but the reporting and handling processes should be more effective. This study deepened the understanding of reporting software's challenges regarding its handling features. The handling processes of incident reports had multiple shortcomings, which may also negatively affect reporters' and other health professionals' experiences in report handling. In addition, this study provides novel insights into managers' perceptions of the future development of a real‐time warning system.

## Author Contributions

Saija Koskiniemi: Conceptualisation, Formal analysis, Investigation, Data curation, Writing – original draft, Writing – review and editing, Visualisation, Laura Jukarainen: Investigation, Writing – review and editing. Tiina Syyrilä: Conceptualisation, Investigation, Writing – review and editing, Supervision. Elizabeth Manias: Conceptualisation, Writing – review and editing. Katri Hämeen‐Anttila: Conceptualisation, Writing – review and editing, Supervision. Marja Härkänen: Conceptualisation, Investigation, Writing – review and editing, Supervision.

## Disclosure

Declaration of Generative AI and AI‐Assisted Technologies in the Writing Process: During the preparation of this work, the authors used Grammarly in order to check grammar and spelling of the text. After using this tool, the authors reviewed and edited the content as needed and take full responsibility for the content of the publication.

## Conflicts of Interest

The authors declare no conflicts of interest.

## Supporting information


**Data S1:** COnsolidated criteria for REporting Qualitative research (COREQ) Checklist.

**Data S2:** The discussion guide.

## Data Availability

Data from this study may be shared upon reasonable request.
