# Supplementary material for: Nurse and Other Healthcare Managers' Experiences and Recommendations for Patient Incident Reporting Processes and Real‐Time Software Development: A Qualitative Study
Source: J Adv Nurs. 2025 Sep 18;82(6):6349–61. doi: 10.1111/jan.70220 (PMC13176687; doi:10.1111/jan.70220)
Supplement: Supplementary file 2 — Data S2: The discussion guide. [file JAN-82-6349-s002.docx]

**Supplementary Material II.** The discussion guide.

| **Theme 1. Beginning**  The interviewer and observer introduce themselves, including their names, current job titles, and working units. The study’s background (SK’s doctoral research, part of a broader research project) is stated. The introduction outlines the structure of the discussion and the roles of the researchers within it.   1. Could you please tell us your first name and working unit as precisely as you want to tell it? |
| --- |
| **Theme 2. The reporting software**   1. What handling features of the incident reporting software are you satisfied with, and why? 2. What handling features of the incident reporting software are you dissatisfied with, and why? 3. Please describe what the best and most functional reporting software would be like. 4. Imagine you have one minute to inform the decision-makers about the incident reporting software’s handling features that require development. What would you like to say?   **The next slide, including the following texts, is shown to the participants:**   - What are your opinions of the following things, considering the development of patient safety incident reporting software handling features: - Classification and answer options on the reporting form - Number of fields to fill in - Ready-made reports/statistics from the system - Report transmission to the correct handler - Forwarding reports to another handler or another unit - Anonymity of the reports - Software technical handiness while handling reports   **Participants are asked to discuss things shown on the slide.** |
| **Theme 3. Processing reports**   1. How does the incident report handling process work in your organisation? 2. How would you modify your organisation’s incident report handling process? 3. How could the staff’s experience with incident report handling be improved? |
| **Theme 5. Possible future developments in reporting software**   1. Imagine having a real-time alarm system in the patient safety incident software. What things would you like the alarm system to alert you to in real-time? 2. What considerations should be taken into account when developing a real-time alarm system from the perspectives of the client, patient, personnel, and organisation? |
| **Theme 6. Closing**   1. Is there anything else you would like to add to the discussion that has taken place?   The interviewer summarises the main points of the discussion and ensures that the participants confirm the accuracy of the summary. |
